# Enterobacterial Small Mobile Sequences Carry Open Reading Frames and are Found Intragenically—Evolutionary Implications for Formation of New Peptides

**Published:** 2007-10-16

**Authors:** Nicholas Delihas

**Affiliations:** Department of Molecular Genetics and Microbiology, School of Medicine, SUNY, Stony Brook, NY 11794-5222, U.S.A

**Keywords:** intergenic repeat units, mobile elements, intragenic insertions, protein domains, gene evolution

## Abstract

Intergenic repeat units of 127-bp (RU-1) and 168-bp (RU-2), as well as a newly-found class of 103-bp (RU-3), represent small mobile sequences in enterobacterial genomes present in multiple intergenic regions. These repeat sequences display similarities to eukaryotic miniature inverted-repeat transposable elements (MITE). The RU mobile elements have not been reported to encode amino acid sequences. An in silico approach was used to scan genomes for location of repeat units. RU sequences are found to have open reading frames, which are present in annotated gene loci whereby the RU amino acid sequence is maintained. Gene loci that display repeat units include those that encode large proteins which are part of super families that carry conserved domains and those that carry predicted motifs such as signal peptide sequences and transmembrane domains. A putative exported protein in *Y. pestis* and a phylogenetically conserved putative inner membrane protein in *Salmonella* species represent some of the more interesting constructs. We hypothesize that a major outcome of RU open reading frame fusions is the evolutionary emergence of new proteins.

## Introduction

A 127-bp repeat unit, enterobacterial repetitive intergenic consensus (ERIC), termed RU-1 in this work, was previously characterized and found in multiple intergenic regions of genomes of Enterobacteriaceae (Sharples and Lloyd, 1990; Hulton et al. 1991; Bachellier et al. 1999; De Gregorio et al. 2005; Wilson and Sharp, 2006). Another class of mobile repeat units of approximately 167-bp, *Yersinia* palindromic sequences (YPAL) and termed RU-2 in this study, was recently reported to be present in *Yersinia* species (De Gregorio et al. 2006). Independently, we also detected this class of repeat unit, which has an approximate 168-bp chain length and is found in 60 different intergenic regions of the *Yersinia pestis* chromosome. A third mobile element (RU-3) of 103-bp was found in *E. coli* and is reported here for the first time. These fascinating mobile sequences are part of a class of bacterial small repeat elements that have close similarities to miniature inverted-repeat transposable elements (MITE) of eukaryotes and archaea microorganisms (Bureau and Wessler, 1992; Redder et al. 2001) in terms of having small polynucleotide chain lengths and terminal inverted repeats. The enterobacterial repeat elements also display stable stem loop secondary structures, TA dinucleotides at their terminal ends and TA duplications at target site insertions (De Gregorio et al. 2006; Wilson and Sharp, 2006). Neither bacterial, archaeon, or eukaryotic mobile elements encode proteins and thus are considered non-autonomous transposable elements. Although the mechanism of transfer has not been elucidated, bacterial repeat units may be moved within genomes by transposases similar to the model for eukaryotic MITE mobility (Feschotte et al. 2005).

Here we employ in silico methods and show that the three enterobacterial repeat sequences display open reading frames which are found fused, in frame, with other open reading frames such that the repeat unit amino acid sequence is maintained. The presence of open reading frame fusions in gene loci of Enterobacteriacae has not been previously reported (Sharples and Lloyd, 1990; Hulton et al. 1991; Lupski and Weinstock, 1992; Bachellier et al. 1999; Claverie and Ogata, 2003; De Gregorio et al. 2005; De Gregorio et al. 2006). However, repeat elements have been detected intragenically in *Rickettsia* species (Ogata et al. 2000) and overlap of repeat nucleotide sequences in some flanking genes in enterobacteria has been reported (Wilson and Sharp, 2006). We discuss the possible evolutionary significance of repeat element fusions in light of François Jacob’s far-sighted evolutionary “tinkering” concept of random fusion of motifs and the formation of new genes (Jacob, 1977; Jordan, 2006).

## Materials and Methods

Alignment of nucleotide and amino acid sequences was by the DNASTAR, Inc. MegAlign program. Parameters used were either ClustalV, with gap penalty 10, gap length 10; or ClustalW with gap penalty 15, gap length, 6.66. The percent sequence identities were based on programs from DNA-STAR. Alignment of some nt sequences was by LALIGN (Huang and Miller, 1991). LALIGN is useful in some nt sequence comparisons in that it allows for local, global and global without end-gap penalty alignments between two sequences.

The Swiss Institute of Bioinformatics SIB ExPASy translate tool was used to find open reading frames and frame shift amino acid sequences from nucleotide sequences.

To search for repeat nt sequences in genomes, NCBI and DOE Joint Genome Institute Blast servers were used. To search for genomic loci that have similarities to RU aa sequences, the SIB ExPASy Blast server was used.

To find predicted protein transmembrane and signal peptide sequence motifs, the Center for Biological Sequence Analysis TMHMM-2.0 (Krogh et al. 2001) and SignalP servers (Bendtsen et al. 2004) as well as the “DAS” Transmembrane prediction server (Cserzo et al. 1997) were used. To find other predicted motifs/domains, the Welcome Trust Sanger Institute Pfam and EMBL-EBI InterProScan Sequence Search servers were used.

RNA secondary structure modeling of RU nt sequences was performed with the Zuker and Turner Mfold, version 3.2 program (Zuker, 2003). Standard constraint parameters were used: maximum interior/bulge loop size was 30, maximum asymmetry of an interior/bulge loop was 30 and there was no limit on maximum distance between paired bases.

Random nucleotide sequences were generated by the Molbiol.ru Random Nucleotide Sequence program.

## Results

### RU-1 repeat sequences

The *E. coli* consensus 127-bp RU-1 sequence (Wilson and Sharp, 2006) was employed to scan bacterial genomes via a genomic blastn search. As previously described (Sharples and Lloyd, 1990; Hulton et al. 1991), multiple repeats are found in intergenic regions of enterobacteria. However, the reverse complement of the 127-bp RU-1 sequence is found to have an open reading frame that translates to a 42 aa peptide (Table 1a). To search genomes for RU-1 type sequences, a related repeat sequence (SeRU-1) found in *Salmonella enterica subsp. enterica serovar Choleraesuis str. SC-B67*, has been used as a reference guide (Table 1a). The SeRU-1 nt sequence was chosen because it is found to be particularly abundant in intergenic region of *Erwinia carotovora subsp. atroseptica SCRI1043*. It also displays a 42 translated amino acid sequence (Table 1a). In addition, a frame shift of the SeRU-1 (termed SeRU-1f) also has an open reading frame and consists of a 41 aa sequence (Table 1a). By utilizing protein database BLAST searches (Gasteiger et al. 2003), RU amino acid sequences were found fused to other open reading frames in annotated gene loci (Tables 2 and 3).

**Figure 1:**
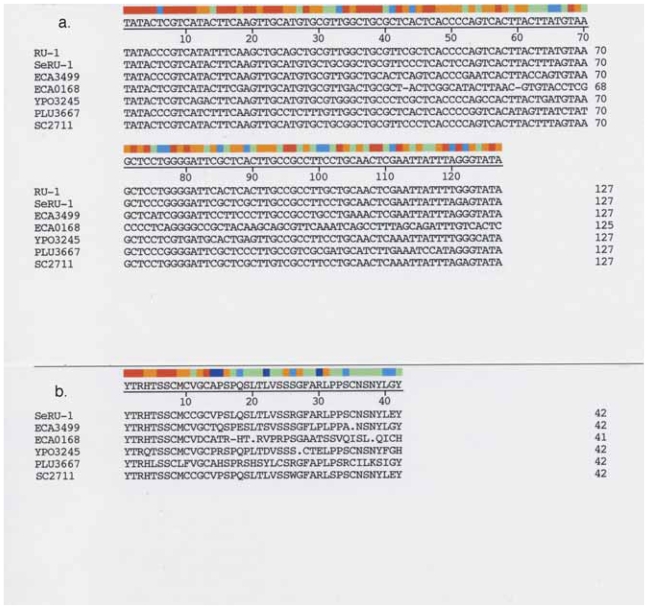
(**a**) Alignment of nt sequences from gene loci *ECA3499*, *ECA0168*, *YPO3245*, *PLU3667*, and *SC2711* with repeat element nt sequences from RU-1 and SeRU-1. Only partial nt sequences of loci are shown. The top sequence represents the consensus sequence and the color bar reflects degree of similarity at each position. Alignment was by the DNASTAR ClustalW program and parameters used as described in Materials and Methods. (**b**) Alignment of amino acid sequences of translated open reading frames from *ECA3499*, *ECA0168*, *YPO3245*, *PLU3667*, *SC2711* and the translated amino acid sequence of SeRU-1.

Figure 1a shows an alignment of SeRU-1 type nucleotide sequences found in annotated gene loci and Figure 1b the SeRU-1 related amino acid sequences present in these loci. Gene loci shown represent large proteins that have conserved domains (*ECA3499*, amidase and *ECA0168*, L-threonine 3-dehydrogenase), unique open reading frames displaying sequence similarities to conserved motifs (*YPO3245* hypothetical protein with PapB motif and *PLU3667* hypothetical protein, HlyD motif), and a hypothetical protein with no similarities to known motifs (*SC2711*). For a reference guide, the consensus repeat RU-1 sequence and the SeRU-1 in *S. enterica* were used.

### *E. carotovora, ECA3499* (amidase)

**Figure 2:**
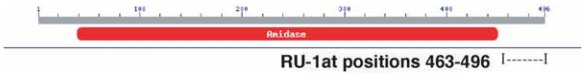
Diagrammatic representation of *ECA3499* amino acid chain showing the amidase conserved domain (Geer et al 2002) (as shown on the NCBI web site: http://www.ncbi.nlm.nih.gov/BLAST/Blast.cgi). The region of the peptide chain containing the RU-1-like sequence (positions 463–496) is also shown.

*ECA3499* encodes a predicted 496 amino acid amidase in *Erwinia carotovora subsp. atroseptica SCRI1043* (Bell et al. 2004). The *ECA3499* sequence displays a conserved amidase domain that starts at amino acid position 30 and ends at position 450 (Geer et al. 2002). The amidase sequence is highly conserved across bacterial genera, however the peptide chain lengths in other species analyzed are uniformly shorter than the *ECA3499* protein length where the C-terminal end is at amino acid position 461 (*ECA3499* terminates at aa position 496). This is due to addition of RU-1 to the 3′-end of *ECA3499*. Fusion of RU-1 with the *ECA3499* nt sequence appears to have eliminated the normal stop codon and the RU is located at a position on the amino acid chain which is beyond the conserved domain (Fig. 2). The RU-1 nt sequence and nucleotide positions 1387–1491 of *ECA3499* are highly similar (Fig. 1a). The percent identity is greater than 80% (Table 4). Amino acid positions 463–496 of *ECA3499* are also highly similar to the SeRU-1 aa sequence (Fig. 1b). Amidase genes from other species have not been found to contain RU-1 type sequences, e.g. *Yersinia*, *Anabena*, *Pseudomonas* and *E. coli*.

Although the aa sequence of the RU-1 insert in *ECA3499* is eight amino acids shorter than the SeRU-1 aa sequence (Fig. 1b), the comparable nt sequence downstream of the 3′ terminal end of the *ECA3499* coding sequence is highly similar to the RU-1 sequences and is of the identical length of the RU (Fig. 1a). However a point mutation results in creation of a stop codon at aa position 35 (Fig. 1b) which prevents read through of the rest of the RU-1 nt sequence. A hypothetical translation of the nt sequence beyond the C-terminal end amino acid sequence.....LPPA (i.e. beyond the stop codon) gives the sequence NSNYLGY, which compares very favorable with the RU-1 aa sequences in the same location, NSNYLEY (Fig. 1b).

The function of the RU addition to this gene is unknown. However presence of the RU nt sequence at the 3′ terminal region of the gene and beyond may have significance in regulation of the transcript (De Gregorio et al. 2005; Wilson and Sharp, 2006). Related to possible function, the secondary structural model of the RU-1 nt sequence in the *ECA3499* locus and its comparable downstream sequence displays a long stable stem loop (data not shown).

### *E. carotovora, ECA0168* (L-threonine 3-dehydrogenase)

*ECA0168* is predicted to encode a 361 amino acid L-threonine 3-dehydrogenase enzyme in *E. carotovora*. L-threonine 3-dehydrogenase is a highly conserved protein in Gram-negative bacteria and displays the super-family AdhC zinc-binding conserved domain which encompasses positions 163–304 (from Pfam: Search Pfam Server). With the exception of *E. carotovora*, all enterobacterial L-threonine 3-dehydrogenase proteins have a predicted chain length of 341 aa. Thus the *ECA0168* sequence is longer, which is similar to the case of the *ECA3499* amidase gene. This is due to addition of the RU-1 sequence to the 3′ end of the ancestral gene.

Unlike *ECA3499*, the entire RU-1 nucleotide sequence is not represented in the *ECA0168* locus (Fig. 1a). High identity with the RU-1 nt sequence ends at nt position 42 and falls off drastically thereafter. Only 26 of the remaining 83 nt are identical to the RU and the amino acid similarity only spans the N-terminal 13 amino acids of the RU-1 sequence (Fig. 1b).

The RU-1 aa sequence is found at the C-terminal end of L- threonine 3-dehydrogenase at amino acid positions 344–356. The total chain length of the enzyme is 361 aa, thus there are an additional five amino acids at the C-terminal end that are unrelated to the RU-1 aa sequence, i.e. ATRHT_361_. The terminal *ECA0168* sequence containing the RU is _344_YTRHTSSCMCVD-CATRHT_361_ (Fig. 1b). Thus the RU-1 sequence is embedded within the 3′ end of *ECA0168* with unrelated sequences.

### *Y. pestis, YPO3245* (PapB)

The *YPO3245* open reading frame has a translated sequence of 248 aa. This sequence is found completely conserved in strains of *Y. pestis* and *Y. pseudotuberculosis* but has not been detected in other *Yersinia* species and thus appears unique to these organisms. The 248 aa sequence has small but significant similarities to the *E. coli UT189* PapB regulatory protein at positions 144–229 (32% identity, E-value = 9e-05, from Expasy Blast Server). Analysis of the *YPO3245* aa sequence by the Pfam Server shows a predicted PapB domain at positions 191–223. The RU-1 sequence is at aa positions 223–248 of *YPO3245* and therefore is outside of the PapB domain. Here again, the RU1-appears to be fused to a putative domain at the C-terminal end. PapB regulates *pap* genes, which are responsible for P pili assembly in *E. coli*.

Alignment of the *YPO3245* nucleotide sequence including downstream sequences (45 nucleotides) with the SeRU-1 sequence shows that *YPO3245* and its downstream region has the full length nt sequence of the RU (Fig. 1a). Alignment of amino acid sequences with those of SeRU-1 (Fig. 1b) shows that *YPO3245* has a stop codon that truncates the aa chain length (Fig. 1b).

### *P. luminescens, PLU3667* (HlyD)

PLU3667 is an open reading frame in the Photorhabdus luminescens subsp. laumondii TTO1 and is annotated as a hypothetical protein of 145 amino acid chain length. This sequence has similarities to hemolysin (RTX toxin) secretion protein (Duchaud et al. 2003). Blast searches using the ExPASy Server show *plu3667* to have a 40%–50% aa sequence identity to the large family of hemolysin (HlyD) secretion proteins at its C-terminal region at aa positions 79–134. The size of the *PLU3667* open reading frame (145 aa) however is smaller than the HlyD super family of proteins (~471 aa), including the putative HlyD in *P. luminescens* (*PLU0635*). *PLU3667* may have originated by duplication of a segment of the *hylD* gene.

Nucleotide positions 25–148 of *PLU3667* display a high identity with RU-1 sequences, about 74%–79% (Table 4). Figure 1b displays an alignment of amino acid sequences in the RU-1 region. This shows a close similarity of the *PLU3667* aa sequence to the entire SeRU-1aa sequence. The RU-1 like sequence (at aa positions 9–50 of *PLU3667*) is outside of the region that has sequence similarities to HlyD proteins (aa positions 79–134 of *PLU3667*).

*PLU3667* appears to be a fusion between an RU-1 nt sequence at its 5′ end region, and an HlyD type sequence at its 3′ end with an approximate 120nt spacer between them. *PLU3667*, together with *YPO3245* are examples of open reading frames formed by fusion of repeat units with sequences displaying similarities to conserved motifs.

### *S. enterica, SC2711* (hypothetical protein)

*SC2711* is annotated as a 112 amino acids hypothetical protein in *S. enterica subsp. enterica serovar Choleraesuis str. SC-B67*. Amino acid positions 32–73 of *SC2711* show a high similarity to the RU-1 nt sequence (Fig. 1a) with an identity of 96.9% (Table 4).

The overall nt sequence of *SC2711* is highly conserved in related species, e.g. *S. enterica subsp. enterica serovar* Typhi Ty2 (96%), however a locus comparable to *SC2711* has not been annotated in most related strains/species, but its nt sequence is present in the identical chromosomal region. In addition, several strains (*Salmonella enterica subsp. enterica serovar Paratyphi A str. ATCC 9150, S. enterica subsp. enterica serovar Typhi Ty2 and S. enterica subsp. enterica serovar Typhi str CT18*) show disrupted open reading frames and these have sustained base pair changes that result in the presence of several stop codons, thus creating truncated open reading frames. The lack of phylogenetic conservation of this open reading frame suggests that that *SC2711* is not a functional gene locus. The RU open reading frame fusion in *SC2711* may represent a “trial and error fusion” in the proposed process of gene evolution by mobile elements.

### Frame shift sequence (RU-1f)

A frame shift sequence of RU-1 (termed RU-1f), shows a translated 41 amino acid (Table 1a). Unlike the aa sequence of RU-1, RU-1f displays a high number of hydrophobic residues, i.e. 19 out of 41 are hydrophobic amino acids and there are clusters of hydrophobic residues at both the N-terminal region (positions 7–18) and C-terminal region (positions 30–37). This property may facilitate formation of transmembrane domains when fused with other open reading frames.

Table 3 shows loci that contain the RU-1f sequence. Most loci are relatively small but there is a high presence of open reading frames annotated as putative membrane proteins. Three gene loci, *ECA0041*, *ECA4465*, and *STM0083* are annotated as putative membrane proteins. All nine loci listed display one to three predicted transmembrane helices by TMHMM and “DAS” transmembrane prediction servers (Krogh et al. 2001; Cserzo et al. 1997). In addition, *YE2643*, a putative exported protein precursor from *Yersinia enterocolitica subsp. enterocolitica 8081* gives a predicted signal peptide sequence. As most of these open reading frames are small, they may or may not represent functional genes. However the dramatic uniformity in transmembrane domain predictions for all nine loci containing the RU-1f sequence may indicate that the RU-1f sequence can form the basis for development of functional membrane proteins and/or exported proteins with additional genomic rearrangements.

### *S. typhimurium* LT2, *STM0083* (putative inner membrane protein)

**Figure 3:**
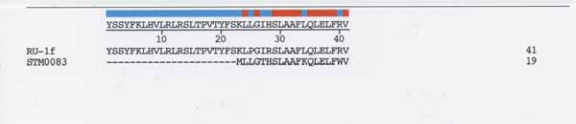
Alignment of amino acid sequences from translated locus *STM0083* with the amino acid sequence RU-1f frame shift sequence. Alignment was by DNASTAR ClustalW.

**Figure 4:**
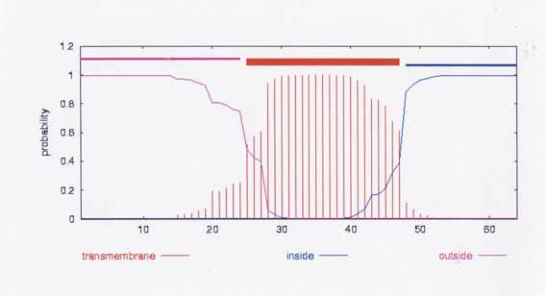
Predicted transmembrane domain from *STM0083* amino acid sequence. Data obtained by TMHMM version 2 (Krogh et al. 2001). The graph is as shown on the Center for Biological Sequence Analysis (CBS) website.

One example from Table 3 will be used to show the relationship of an open reading frame locus with the RU-1f sequence. *STM0083*, an open reading frame encoding a putative inner membrane in *Salmonella typhimurium LT2*, has a high nt sequence identity at its 5′ end with nt positions 67–125 of RU-1f (84.2% identity, using the LALIGN Server (Huang and Miller, 1991) (LALIGN is useful for local alignments between two nt sequences). The amino acid sequence alignment is shown in Figure 3. Amino acid positions 24–41 of RU-1f and positions 2–19 of *STM0083* show an identity of 72%. This segment includes one of the hydrophobic aa clusters on RU-1f. The region of the *STM0083* sequence that is fused to RU-1f also has a cluster of hydrophobic amino acids. The high concentration of hydrophobic amino acids in this fusion appears to result in formation of a transmembrane domain (Fig. 4).

The *STM0083* aa sequence is found in all five *Salmonella* species whose genomes have been sequenced with >90% identity and all have a chain length of 64 aa. Thus, there are no stop codons present in orthologs. This is in sharp contrast to the lack of phylogenetic conservation of the *SC2711* aa chain length in related *Salmonella* species. Conservation of the *STM0083* locus aa sequence suggests that this gene locus may be functional.

### *V. cholerae MAK 757, VC2263* (open reading frame)

A repeat unit related to RU-1f was found in *Vibrio cholerae MAK 757.* A search using the *SCH_0078* aa sequence (*SCH_0078* is an ortholog of *STM0083* in *S. enterica* subsp. enterica serovar Choleraesuis str. SC-B67) yielded a positive hit with a 32 amino acid sequence of a small open reading frame annotated as *VC2263* (accession number NP_231894) from *V. cholerae MAK 757* (56% identity, E-value = 0.023). This sequence is: MSMLMGTHSLAAY-LQLQIVWVYTFTKMALNLL. It is repeated 28 times intergenically in the Vibrio genome with greater than 70% identity and E-value = 0.06 or less. This repeat sequence is found in other *Vibrio* species as well. Thus a variant of the RU-1f-type sequence appears to be present in *Vibrio* species, the most distantly related species of the gamma Enterbacteriacae where the repeat element amino acid sequence has been found. The small *VC2263* open reading frame gives a 0.4 probability for a predicted transmembrane motif.

### Repeat unit RU-2

While scanning genomic sequences in *Yersinia species* for the *E. coli* non-coding RNA gene *itsR* (Vogel et al. 2004), a 168-bp sequence was found which is related to a previously described 167-bp repeat (De Gregorio et al. 2006). The nucleotide sequence of a representative repeat element from the *Y. pestis CO92* genome (termed RU-2) and its location in the genome are shown in Table 1b.

RU-2 also displays a translated open reading frame, which can potentially encode a 56 amino acid sequence (Table 1b). The amino acid sequence is present with 100% identity in all genomes of *Y. pestis* and *Y. pseudotuberculosis*. It is also found with >70% identity in other *Yersinia* species, e.g. *Y. enterocolitica*, and *Y. mollaretii*. RU-2 (aa positions 19–56) has also been detected in *Erwinia carotovora*. Because of its high conservation, RU-2 was used as a reference to scan genomes for insertions.

The RU-2 amino acid sequence was analyzed for predicted structure/function domains. The “DAS”—transmembrane prediction server for prokaryote sequences gave a weak signal for a transmembrane motif at aa positions 45–50 of RU-2. In accordance with this, 50% of the amino acids are hydrophobic with a cluster of hydrophobic amino acids at positions 42–50. As shown below, the RU-2 sequence fused to other open reading frames can form predicted transmembrane domain/signal sequences.

Three examples will be given: (1) fusion of RU-2 with an open reading frame that leads to a signal peptide sequence (*YPO1552*), (2) fusion of RU-2 with a conserved motif (isoleucine patch super family, *YP_0349*), and (3) addition of a small part of the RU-2 nt sequence to the sequence of a large gene (*ECA4072*, ABC transporter ATP-binding protein).

### *Y. pestis, YPO1552* (putative exported protein)

*YPO1552* is annotated as a putative144 amino acid exported protein in *Y. pestis CO92* (Parkhill et al. 2001). The amino acid sequence is conserved 100% in seven other strains as well as in *Y. pseudotuberculosis*, but a segment of the *YPO1552* aa sequence (positions ~55–138) is found in more distantly related species: *Salmonella*, *Vibrio*, and *Burkholderia*.

**Figure 5:**
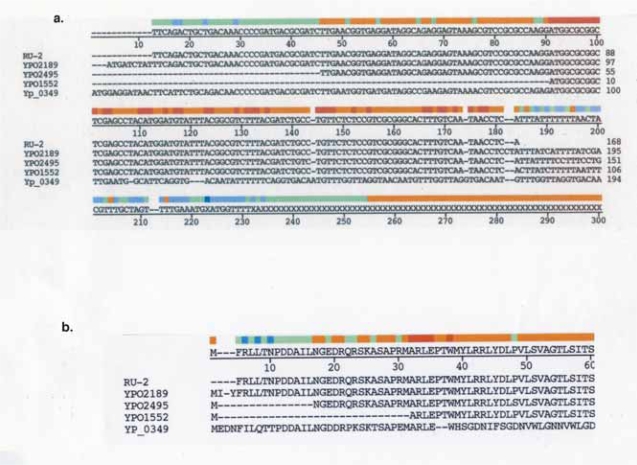
(**a**) Nucleotide sequence alignment of the 168nt nt sequence RU-2 from *Y. pestis CO92* with *Y. pestis CO92* loci *YPO2189*, *YPO2495*, and *YPO1552. YP_0349* is from *Y. pestis biovar Microtus str. 91001*. Only the 5′ nt sequences of loci are displayed. Alignment was by ClustalW. (**b**) Amino acid sequence alignment of RU-2 sequence with N-terminal amino acid sequences from loci *YPO2189*, *YPO2495, YPO1552* and *YP_0349*. Alignment was by ClustalV.

Nucleotide sequence positions in *YPO1552* that encompass RU-2 are 1–90 (Fig. 5a) and amino acid positions 1–30 (Fig. 5b). (It should be noted that in the annotation of the *Y. pestis CO 92* genome, previous reference was made to the presence of a repeat sequences in *YPO1552* (web page: http://www.ncbi.nlm.nih.gov/entrez/viewer.fcgi?db=protein&val=115347304).

**Figure 6:**
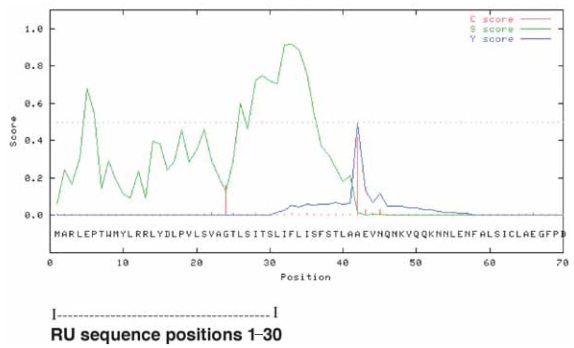
Analysis of aa sequence of the translated *YPO1552* nt sequence as shown by the CBS SignalP 3.0 server (Bendtsen et al. 2004; Emanuelsson et al. 2007). This server predicts signal sequences and signal cleavage sites in the N-terminal regions of peptide chains. Scores are as defined by Nielsen et al. (1997) and refer to C (red), cleavage site score, S-score (green), output from signal peptide networks, and Y-score (blue), which is derived from the C and S scores is presumed to provide the best cleavage site prediction. The position of the RU within the signal sequence is shown in the diagram. The parameters are those set on the server for gram-negative bacteria.

The CBS SignalP server (Bendtsen et al. 2004) shows that the Y*PO1552* amino acid sequence has a high probability of having a signal peptide sequence motif, encompassing positions 1–41 and a cleavage site is predicted to be between positions 41 and 42 (Fig. 6) (signal peptide probability = 0.994; cleavage site probability is 0.989). Thus the RU-2 forms part of a predicted signal peptide sequence.

Similarities of the *YPO1552* sequence with aa sequences in *Salmonella*, *Vibrio*, and *Burkholderia* do not overlap with the RU-2 sequence of *YPO1552*. Of importance, sequences from these three genera all show predicted signal sequences as well, e.g. the *Vibrio sp. DAT722* hypothetical protein sequence give a signal peptide probability of 0.999. However, signal sequences from these species are unrelated to RU-2.

The RU-2 also participates in predicted motifs in other open reading frames annotated in *Y. pestis CO92*. In *YPO2189*, amino acid positions 37–56 of RU-2 overlap a transmembrane domain. *YPO2495* also displays a transmembrane domain region, but in addition, a predicted signal sequence as well (probability = 0.722). Thus RU-2 appears to form predicted structure/function motifs when found fused to several different open reading frames.

### *Y. pestis, YP_0349* (wbbJ)

**Figure 7:**
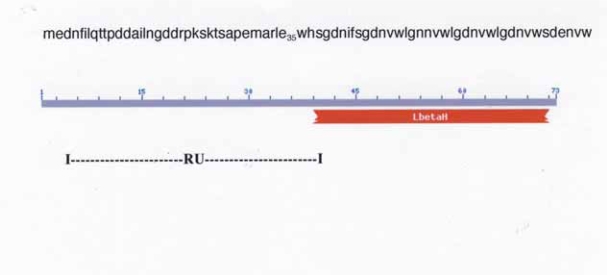
Amino acid sequence of YP_0349 and domain LbetaH (Left-Handed Parallel beta-Helix) location. The RU spans the first half of the peptide chain is shown. A number of bacterial transferases display the LbetaH motif with tandem hexapeptide repeats (Raetz and Rod-erick, 1995). Part of the drawing is from the NCBI web site: http://www.ncbi.nlm.nih.gov/BLAST/Blast.cgi

*YP_0349* (*wbbJ*), annotated as an acetyltransferase, is found in *Y. pestis biovar Microtus str. 91001*. The carboxyl half of the putative peptide chain, starting at position 36 contains hexapeptide repeats, which are characteristic of the isoleucine patch super family and consist of Left-Handed Parallel beta-Helix (LbetaH) (Raetz and Roderick, 1995). The N-terminal sequence, amino acid positions 4–35 has a sequence highly similar to the N-terminal sequence of RU-2 (Fig. 5b). Positions 1–31 of RU-3 and positions 5–35 of *YP_0349* share a 74% identity. The *YP_0349* sequence similar to RU-2 does not include the RU-2 C-terminal cluster of hydrophobic amino acids which, when combined with other sequences produces predicted structure/function domains. The 5′ segment of the RU-2 nt sequence appears inserted into the genome adjacent to a hexapeptide repeat sequence resulting in approximately half of the *YP_0349* peptide chain (N-terminal) consisting of the RU and the C-terminal half containing the hexapeptide repeats (Fig. 7). This is another example of fusion of a repeat element with an open reading frame containing a conserved motif.

### *E. carotovora, ECA4072* (ABC transporter ATP-binding protein)

A search of the *E. carotovora* genome for the RU-2 sequence showed a hit for the 269 aa *ECA4072* (ABC transporter ATP-binding protein) gene. Only a small segment of the 3′ end of RU-2 nt sequence (16 nucleotides) overlaps the 3′ end of the ABC transporter ATP-binding protein coding region, however neither the RU-2 aa sequence, or frame shift aa sequences are present in *ECA4072*. The 16 nt overlap extends the *ECA4072* C-terminal amino acid sequence by 4 amino acids (ERMG_269_). This extension is outside of the ABC super family transporter domain but the extended sequence is not found in orthologs of the ABC transporter gene. Thus this is another example of fusion of an RU sequence at the 3′ end of a gene that is part of a super family. But in this case, the RU nt sequence is present but neither the RU aa sequence or frame shift aa sequences are represented.

### RU-3

The RU-3 nt sequence (Table 1b) is a new repeat element reported here and was found at the 3′ side of the pepB gene in *E. coli O157:H7 EDL933 strain: EDL933*. There are multiple repeats of this sequence and smaller segments found in *E. coli*, *Salmonella*, and *Shigella* with >80% nt sequence identity. However the sequence has not been found in other species. RU-3 shows a long stable stem loop secondary structure with a delta G = −44.2 kcal/mol but displays imperfect inverted repeats. RU-3 carries a 34 amino acid open reading frame (Table 1b). In *E. coli O157:H7 EDL933 strain: EDL933*, there are 22 repeats found in intergenic regions with greater than 58% aa sequence identity and an E-value = 3e-04 or better and smaller segments are found repeated 29 times intergenically.

Seven open reading frames are found to contain segments of the RU-3 sequence (Table 5). These open reading frames consist largely of small to moderate sized hypothetical proteins, which display no domains/signatures. The largest is *c3518* annotated as a hypothetical 152 aa protein in *Escherichia coli CFT073*. The RU-3 sequence is situated in the first third of the *c3518* open reading frame. The aa sequence identity is 74%. *c3518* shows no predicted domains/motifs and there appear to be no orthologs to this open reading frame.

A frame shift of the reverse complement of RU-3 yields a 31 aa sequence (Table 1b). This aa sequence is found in several hypothetical proteins of *E. coli*, but importantly, in a 170 aa putative protein annotated as LivJ. The sequence similarity is between aa positions 12–31 of RU-3f and aa positions 151–170 at the C-terminal end of LivJ:

_12_QFVEF—AILCRPDKAFTPHPA_31_

_151_QFTDFFGS—CRPDKAFTPHPA_170_

LivJ has a high identity (86%, E-value = 7e-12) to part of a highly conserved acyltransferase motif at the N-terminal end (LivJ positions 1–45) but the entire sequence of LivJ yields no orthologs. LivJ appears to be a fusion of part of the conserved acyl-transferase motif at its N-terminal end, a 106 aa spacer and the RU-3f amino acid at its C-terminal end.

## Discussion

We show here that two repeat elements and a newly found repeat sequence in enterobacteria display open reading frames. These elements are found fused to other open reading frames in 24 annotated enterobacterial gene loci. Together with frame shift translated sequences, the RU mobile elements provide different amino acid sequences, which carry different properties for potential fusion to other open reading frames. In all but one of the examples shown, the amino acid sequence of the RU is maintained. RU elements are found fused to several large super family protein genes but are primarily found in other loci, whose translated sequences display similarities to protein conserved domains or form predicted signal sequence and/or transmembrane motifs. Some loci (*PLU3667* and LivJ) appear to have formed by fusion of three units, a conserved domain, spacer sequence and the RU. Open reading frames, which are unique to certain species and not found in closely relates species (e.g. PapB-like sequence found only in *Y. pseudotuberculosis* and *Y. pestis* and not in other *Yersinia* species), may represent recent fusions in evolutionary time.

Several gene loci show that the repeat element aa sequence contributes to structure/function motifs. For example, the RU-2 sequence forms part of a predicted signal peptide sequence in the putative exported protein encoded by *YPO1552* in *Y. pestis*. This is one of the more interesting loci and its potential protein product should be tested experimentally. Also of interest is the phylogenetic conservation of a gene locus sequence in *Salmonella*, *STM0083* that potentially encodes a putative inner membrane protein. This suggests the conserved locus may be functional.

Sequences containing motifs such as signal peptide and transmembrane domains can be potentially useful to a bacterial pathogen. Open reading frames that have these motifs can be looked at as possible “building blocks”, whereby combined with additional open reading frames, may form new toxins that can be secreted or form additional membrane proteins. Frame shift RU-1f fusions may constitute such “building blocks”.

No repeat elements have been found within conserved domain sequences of proteins that are members of super families. Instead, RU fusions are at C-terminal regions where perhaps they act as regulators or have a neutral effect. These domains are ancient and extremely well conserved and thus have evolved optimum sequences for function. Therefore insertion of mobile elements within conserved domains would appear to disrupt function.

Other small repeat elements found in bacteria include the RUP element of *Streptococcus pneumoniae* (Oggioni and Claverys, 1999) and the NEMIS repeat sequences of *Neisseria* sp. (Correia et al. 1988; Mazzone et al. 2001). Both RUP and NEMIS sequences also have open reading frames and are found intragenically, and in *Neisseria* sp., within different classes of transposase genes (data not shown).

Although RU amino acid sequences have not been previously reported to be present intragenically in enterobacteria, the pioneer work of Claverie and co-investigators shows that small repeat sequences specific to the *Rickettsias* are found inserted in protein genes (Ogata et al. 2000; Claverie and Ogata, 2003; Abergel et al. 2006). In eukaryotes, there is evidence for the presence of transposon and retrotransposon sequences in protein gene sequences (Brownell et al. 1989; Smit, 1999; Nekrutenko and Li, 2001; Kazazian, 2004) as well as a particularly interesting transposase sequence fragment found in a new primate gene (Cordaux et al. 2006).

Eukaryotic MITEs have been proposed to have originated from internal deletions of transposons (Feschotte et al. 2005). It is not known how eubacterial RU sequences evolved, although the MITE analogy may pertain. Because of the type of structure/function signatures encompassed by translated sequences, part of RU-1 and RU-2 sequences may have originated from or acquired segments of membrane protein or exported protein genes. The possibility that open reading frames originated from hypothetical random intergenic nucleotide sequences has also been considered. For example, in an analysis of random sequences of 150 nt, one sequence was found to translate to a 49 aa open reading frame. However there are constraints on RU nt sequences in that RUs have stable long hairpin secondary structures and terminal and/or internal inverted repeats. In addition, translation of a set of 150 nt random sequences has not produced open reading frames that are found in cellular proteins (unpublished).

Repeat sequences in intergenic regions of enterobacterial genomes have been proposed to regulate mRNAs encoded by genes that are upstream of the repeat element (De Gregorio et al. 2005; De Gregorio et al. 2006). Interestingly, intergenic repeats elements similar to the RUs of enterobacterial, called CIR motifs, have also been found in *Caulobacter* and these elements may be associated with DNA methylation sites (Chen and Shapiro, 2003). Thus small mobile elements in bacteria may contribute to cell metabolism/molecular functions (intergenic insertions) and possible evolutionary gene development (intragenic insertions).

There is a long-standing interest in genetic repeat elements and their significance in protein gene evolution and several theoretical approaches have been used in an attempt to understand the role of repeat sequences and domain exchanges in protein gene origins (Ohno et al. 1984; Ohno, 1987; Dwyer, 1998; Shiba et al. 2003; Britten, 2006). Insertion of mobile elements into genomes has been referred to as “tinkering” (Jordan, 2006). François Jacob originally proposed the concept of “tinkering” in evolution and that “Evolution works on what already exists ..... combining several systems to produce a more elaborate one” (Jacob, 1977). The RU fusions described here may be part of an evolutionary process of “tinkering” in formation of new proteins.

## Web Sites and Web Pages

Center for Biological Sequence Analysis: CBS: http://www.cbs.dtu.dk/services/TMHMM-2.0/

CBS: http://www.cbs.dtu.dk/services/SignalP/

ch.EMBnet.org: http://www.ch.embnet.org/software/LALIGN_form.html

“DAS”—Transmembrane Prediction server http://www.sbc.su.se/%7Emiklos/DAS/

DNASTAR, Inc: http://www.dnastar.com/

DOE Joint Genome Institute: http://img.jgi.doe.gov/cgi-bin/m/main.cgi/

EMBL-EBI Data Base: http://www.ebi.ac.uk/InterProScan/

Mfold: http://www.bioinfo.rpi.edu/applications/mfold/rna/form1.cgi/

Molbiol.ru: http://molbiol.ru/eng/scripts/01_16.html

National Center for Biotechnology Information (NCBI) GenBank sites: http://www.ncbi.nlm.nih.gov/BLAST/

http://www.ncbi.nlm.nih.gov/gquery/gquery.fcgi/

http://www.ncbi.nlm.nih.gov/Structure/lexington/lexington.cgi?cmd=rps

Swiss Institute of Bioinformatics SIB ExPASy Proteomics Servers: http://www.expasy.ch/

http://www.expasy.org/tools/blast/

http://www.expasy.org/tools/dna.html

The Welcome Trust Sanger Institute Pfam: http://www.sanger.ac.uk/Software/Pfam/search.shtml

The Welcome Trust Sanger Institute Y. enterocolitica *Blast Server*: http://www.sanger.ac.uk/cgi-bin/blast/submitblast/y_enterocolitica/

## Figures and Tables

**Table 1a:** Nucleotide and translated amino acid sequences of repeat elements.

	Genome position
*E. coli127nt RU-1 nucleotide sequence	
5′tatacccgtcatatttcaagctgcagctgcgttggctgcgttcgctcaccccag tcacttacttatgtaagctcctggggattcactcacttgccgccttgctgcaactcg aattattttgggtata3′	
*E. coli127nt RU-1 translated amino acid sequence	
YTRHISSCSCVGCVRSPQSLTYVSSWGFTHLPPCCNSNYFGY	
**S. enterica127nt SeRU-1 nucletotide sequence	
5′tatactcgtcatacttcaagttgcatgtgctgcggctgcgttccctcactccagtc acttactttagtaagctcccggggattcgctcgcttgccgccttcctgcaactcga attatttagagtata3′	2654778–2654904
**S. enterica127 nt SeRU-1 translated amino acid sequence	
YTRHTSSCMCCGCVPSLQSLTLVSSRGFARLPPSCNSNYLEY	
**S. enterica125nt SeRU-1f frame shift nucleotide sequence	
5′tactcgtcatacttcaagttgcatgtgctgcggctgcgttccctcactccagtca cttactttagtaagctcccggggattcgctcgcttgccgccttcctgcaactcgaattat ttagagtata3′	2654780–2654904
**S. enterica125nt SeRU-1f frame shift translated amino acid sequence	
YSSYFKLHVLRLRSLTPVTYFSKLPGIRSLAAFLQLELFRV	

* Reverse complement of sequence shown by Wilson and Sharp (2006). This is a consensus nt sequence that shows a translated amino acid sequence.

** Salmonella enterica subsp. enterica serovar Choleraesuis str. SC-B67.

**Table 1b:** Nucleotide and translated amino acid sequences of repeat elements.

	Genome position
***Y. pestis*CO92 168nt RU-2 nucleotide sequence**	
5′ttcagactgctgacaaaccccgatgacgcgatcttgaacggtgagg ataggcagaggagtaaagcgtccgcgccaaggatggcgcggctcg agcctacatggatgtatttacggcgtctttacgatctgcctgttctctccg tcgcgggcactttgtcaataacctca3′	1009572–1009739
***Y. pestis*CO92 168nt RU-2 translated amino acid sequence**	
FRLLTNPDDAILNGEDRQRSKASAPRMARLEPTWMYLRRLYDLPV LSVAGTLSITS	
*E. coli103nt RU-3 nucleotide sequence	
5′tatgccggatgcggcgtaaacgccttatccggcctacaaagtattgcaaa ttcaacaaattgtgaatcccttgtaggcctgataagtatggcgcatcaggcac3′	3445561–3445459
*E. coliRU-3 translated amino acid sequence	
YAGCGVNALSGLQSIANSTNCESLVGLISMAHQA	
*E. coliRU-3f 3′5′ frame shift translated amino acid sequence	
CAILIRPTRDSQFVEFAILCRPDKAFTPHPA	

* Escherichia coli O157:H7 EDL933.

**Table 2:** Annotated loci containing SeRU-1 amino acid sequence*.

Annotated locus/species	E-value	aa length
*SC2711* hypothetical protein *S. enterica subsp. Enterica serovar Choleraesuis str. SC-B67*	3e-24	112
*P71284* hypothetical protein *E. coli*	1e-17	103
*ECA3499*, probable amidase *E. carotovora subsp. Atroseptica SCRI1043*	2e-09	496
*YPO3245* (contains PapB-like motif) *Y. pestis CO 92, other Y. pestis strains, Y. pseudotuberculosis*	2e-04	248
*PLU*3667 hypothetical protein (similarities with hemolysin) *P. luminescens subsp. Laumondii TTO1*	8e-04	145
*YPO0307* hypothetical protein *Y. pestis CO 92*	0.060	84
*ECA*0168 L-threonine 3-dehydrogenase *E. carotovora subsp. Atroseptica SCRI1043*	0.14	361
*ECA1899* hypothetical protein	0.35	126

* Data from ExPASy Blast Server.

**Table 3:** Annotated Enterobacterial loci containing frame-shift RU-1f amino acid sequence*.

Annotated locus/species	E-value	aa length
*ECA3326* hypothetical protein *E. carotovora subsp. atroseptica SCRI1043*	1e-16	55
*ECA0041* putative membrane protein *E. carotovora subsp. atroseptica SCRI1043*	2e-05	75
*YE3156* hypothetical protein *Yersinia enterocolitica subsp. enterocolitica 8081*	1e-04	162
*YE2643* putative exported protein precursor *Yersinia enterocolitica subsp. enterocolitica 8081*	0.008	66
*ECA4465* putative membrane protein *E. carotovora subsp. atroseptica SCRI1043*	0.019	96
*STM0083* putative inner membrane protein *S. typhimurium LT2*	0.081	64
*c4993* hypothetical protein *E. coli CFT073*	0.11	109
*YPO0143* hypothetical protein *Y. pestis CO92*	0.19	64
plu0725 hypothetical protein *P. luminescens subsp. laumondii TTO1*	2.0	101

* Data from Blast with ExPASy Blast Server.

**Table 4:** Nucleotide sequence identity.

Percent identity		
	RU-1	SeRU-1
*ECA3499*	82.7	81.1
*ECA0168*	48.8	48.0
*YPO3245*	85.0	81.1
*PLU3667*	78.8	74.0
*SC2711*	85.8	96.9

**Table 5:** Annotated Enterobacterial loci containing RU-3 amino acid sequence*.

Annotated locus/species	E-value	aa length
*c0014* hypothetical protein *Escherichia coli CFT073*	1e-12	45
*c3518* hypothetical protein *Escherichia coli CFT073*	7e-11	152
*c4359* hypothetical protein *Escherichia coli CFT073*	8e-09	123
*UTI89_C2441* hypothetical protein *Escherichia coli*	2e-06	140
*ECP_0131* hypothetical protein *Escherichia coli 536*	1e-08	60
*yahH* putative uncharacterized protein *Escherichia coli*	3e-07	106
Hypothetical protein *Escherichia coli*	0.011	92

* Data from Blast with ExPASy Blast Server.

## Abbreviations

RU: repeat unit
nt: nucleotide
aa: amino acid
bp: base pair
ERIC: enterobacterial repetitive intergenic consensus
YPAL: Yersinia palindromic sequences
MITE: miniature inverted-repeat transposable elements
